# Rational design of photocatalysts for ammonia production from water and nitrogen gas

**DOI:** 10.1186/s40580-021-00273-8

**Published:** 2021-08-02

**Authors:** Seokwoo Choe, Sung Min Kim, Yeji Lee, Jin Seok, Jiyong Jung, Jae Sung Lee, Youn Jeong Jang

**Affiliations:** 1 Department of Chemical Engineering, Hanyang University, 222 Wangsimni-ro, Seongdong-gu, Seoul, 04763 Republic of Korea; 2 Department of Energy and Chemical Engineering, Ulsan National Institute and Science and Technology, 50, UNIST-gil, Ulsan, 44919 Republic of Korea

**Keywords:** Photocatalyst, Ammonia, Nitrogen

## Abstract

Photocatalytic N_2_ reduction has emerged as one of the most attractive routes to produce NH_3_ as a useful commodity for chemicals used in industries and as a carbon-free energy source. Recently, significant progress has been made in understanding, exploring, and designing efficient photocatalyst. In this review, we outline the important mechanistic and experimental procedures for photocatalytic NH_3_ production. In addition, we review effective strategies on development of photocatalysts. Finally, our analyses on the characteristics and modifications of photocatalysts have been summarized, based on which we discuss the possible future research directions, particularly on preparing more efficient catalysts. Overall, this review provides insights on improving photocatalytic NH_3_ production and designing solar-driven chemical conversions.

## Introduction

Recently, there has been an increasing focus on ammonia (NH_3_) as not only a key commodity for chemicals widely used in industries [1, 2], but also as a liquid energy carrier [3] that enables transport and supply of hydrogen (H_2_) gas through cracking. Furthermore, NH_3_ is an alternative carbon-free energy source that can be utilized in energy-conversion devices, for example, direct NH_3_ fuel cells [4, 5]. Traditionally, NH_3_ production has mainly relied on the Haber–Bosch process, which is energy intensive and generates considerable volume of CO_2_ into the atmosphere because of these requirements, along with the needs for H_2_ and the extreme operation condition [6].

Photochemical NH_3_ production using N_2_ and water as a hydrogen source at ambient temperature and pressure can offer a promising alternative route that is less energy intensive and reduces CO_2_ emission, thereby significantly reducing environmental concerns [7]. NH_3_ production paired with water oxidation is a thermodynamically uphill reaction; therefore, external energy input is necessary [8]. Photocatalysts can utilize solar energy, which is supplied to Earth with sufficient solar power and abundance and facilitate NH_3_ production. Since TiO_2_ photocatalysts applied to N_2_ reduction, new photocatalysts and their modifications have been intensively suggested to improve photocatalytic NH_3_ production [9].

Major advances in metal oxides such as TiO_2_, WO_3_, and SrTiO_3_, bismuth oxyhalides, and polymeric carbon nitride photocatalysts have resulted in interests in the development of photocatalysts and their applications for NH_3_ production [10–14]. This review is composed of mainly two sections: (1) fundamentals for photocatalytic NH_3_ production, (2) strategies to develop photocatalysts. The first section focuses on the principles and mechanisms involved in the overall photocatalytic reaction. The second section contains recent progresses in the development of catalysts and their major advantages with regard to catalytic performance. The insights and discussions provided in this review will serve as useful resources for further development of photocatalysts and provide future research direction for photocatalytic NH_3_ production.

## Fundamentals for photocatalytic NH_3_ production

### Principle and mechanism

NH_3_ production by N_2_ reduction (N_2_(g) + 6H^+^  + 6e^−^ ⇌ 2NH_3_(g), E^0^ = 0.092 V vs. SHE) paired with O_2_ production by water oxidation (2H_2_O(l) ⇌ O_2_(g) + 4H^+^  + 4e^−^, E^0^ = 1.229 V vs. SHE) is a thermodynamically uphill reaction that requires a potential of at least 1.137 eV [15] per electron [16]. Moreover, three electrons are required to produce an NH_3_ molecule, and four holes are required to produce an O_2_ molecule according to eqns. (1) and (2):

$${\text{Absorption of light }}:{\text{photocatalyst}} + hv{ }\left( { > E_{{\text{g}}} } \right) \to e_{{{\text{CB}}}}^{ - } + {\text{h}}_{{{\text{VB}}}}^{ + }$$

1 $${\text{Nitrogen reduction }}\left( {{\text{NRR}}} \right) : {\text{N}}_{2} + 6{\text{H}}^{ + } + 6{\text{e}}_{{{\text{CB}}}}^{ - } \to 2{\text{NH}}_{3}$$

2 $${\text{Water oxidation }}\left( {{\text{OER}}} \right){ }:{ }2{\text{H}}_{2} {\text{O}} + 4{\text{h}}_{{{\text{VB}}}}^{ + } \to {\text{O}}_{2} + 4{\text{H}}^{ + }$$

**Fig. 1:**
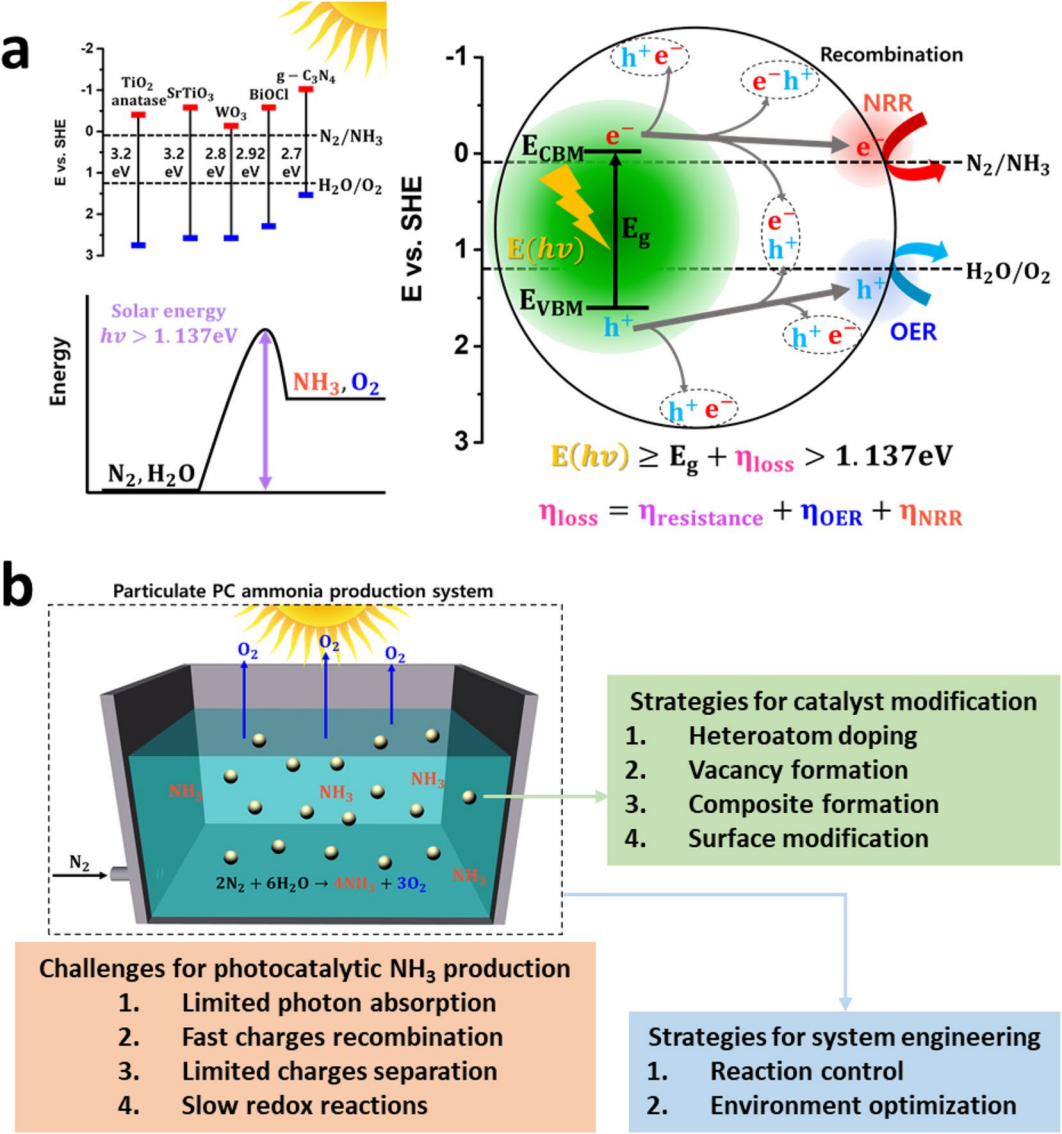
**a** The schematic illustration of photochemical nitrogen reduction reaction [15]. **b** The challenges and strategies for photocatalytic NH_3_ production in particulate photocatalytic (PC) ammonia production system

A simplified schematic diagram for photocatalyic NH_3_ production and challenges in designing photocatalysts are summarized in Fig. 1. For overall NH_3_ production, photons with energies > 1.137 eV are required to generate photoexcited electron–hole pairs that carry out the desired redox reactions at the active sites on the surfaces of the photocatalysts. Thus, a photocatalyst must have an energy band gap, (E_g_ = E_CBM_-E_VBM_, where CBM and VBM represent conduction band minimum and valence band maximum), larger than the energy required for the overall NH_3_ production. In addition, a photocatalyst must have a suitable conduction and valence band alignment to drive two half-reactions using photoexcited electrons (e^−^) and holes (h^+^) under solar light irradiation.

The major factors to consider for photocatalyst designs are (1) photon absorption followed by photoexcited carrier generation, (2) their migration and separation, and (3) their consumption by redox reactions on the surfaces of photocatalysts. The dominant photon energy spectrum obtained from the sunlight is in the range of 390–700 nm. Furthermore, because photocatalytic redox reactions require kinetic overpotentials, photocatalysts have a bandgap energy in the range of 1.6–2.4 eV. The generated photoexcited carriers can migrate to active sites on the surface where redox reactions carried out. However, since the kinetics of carrier recombination in the interiors of the photocatalysts is fast, considerable amounts of photoexcited carriers are recombined [17]. Moreover, photoexcited carriers can be trapped in the interior and surface of the photocatalyst, which causes photocorrosion. Thus, a photocatalyst design to improve electron–hole pair migration and separation is important for further development of photochemical NH_3_ production [18].

Most semiconductors that act as light absorbers in photocatalysts are inactive in the redox reactions with the photoexcited carriers; thus, the active sites on the surface of semiconductor are decorated with cocatalysts [19]. For example, cocatalysts, such as RuO_x_, CoO_x_, and Co-pi [20–22], combined on the surface of the semiconductor for facile water oxidation by providing active sites. Another critical point to note in NH_3_ production via photochemical N_2_ reduction is the facile competitive hydrogen evolution reaction by water reduction. NH_3_ production is kinetically much more complex than water reduction, although the thermodynamic requirements for those reactions are similar [23]. This indicates that photochemical H_2_ production may be predominant, resulting in inactive NH_3_ production. Thus, it is critical to modify the surface that provides active sites for the desired redox reaction using suppressing competitive reactions on the semiconductors.

It is also necessary to note the importance of rate balance for photocatalytic reactions on photocatalysts. Since electrons and holes are generated in pairs under illuminations, their consumption must be coupled as well. For instance, if oxidation reactions involving photogenerated holes are sluggish, the total NH_3_ production rate should be similar. In this case, scavengers, such as ethanol, methanol, and sulfites, can be employed as sacrificial reagents to facilitate oxidation reaction using photoexcited holes, thereby preventing from photoexcited carrier recombination [24]. However, since scavengers may interfere with counter reactions, here N_2_ reduction, or product evaluation methods, the target scavenger should be chosen carefully. It is discussed in detail in the next section.

### Experimental process

**Fig. 2:**
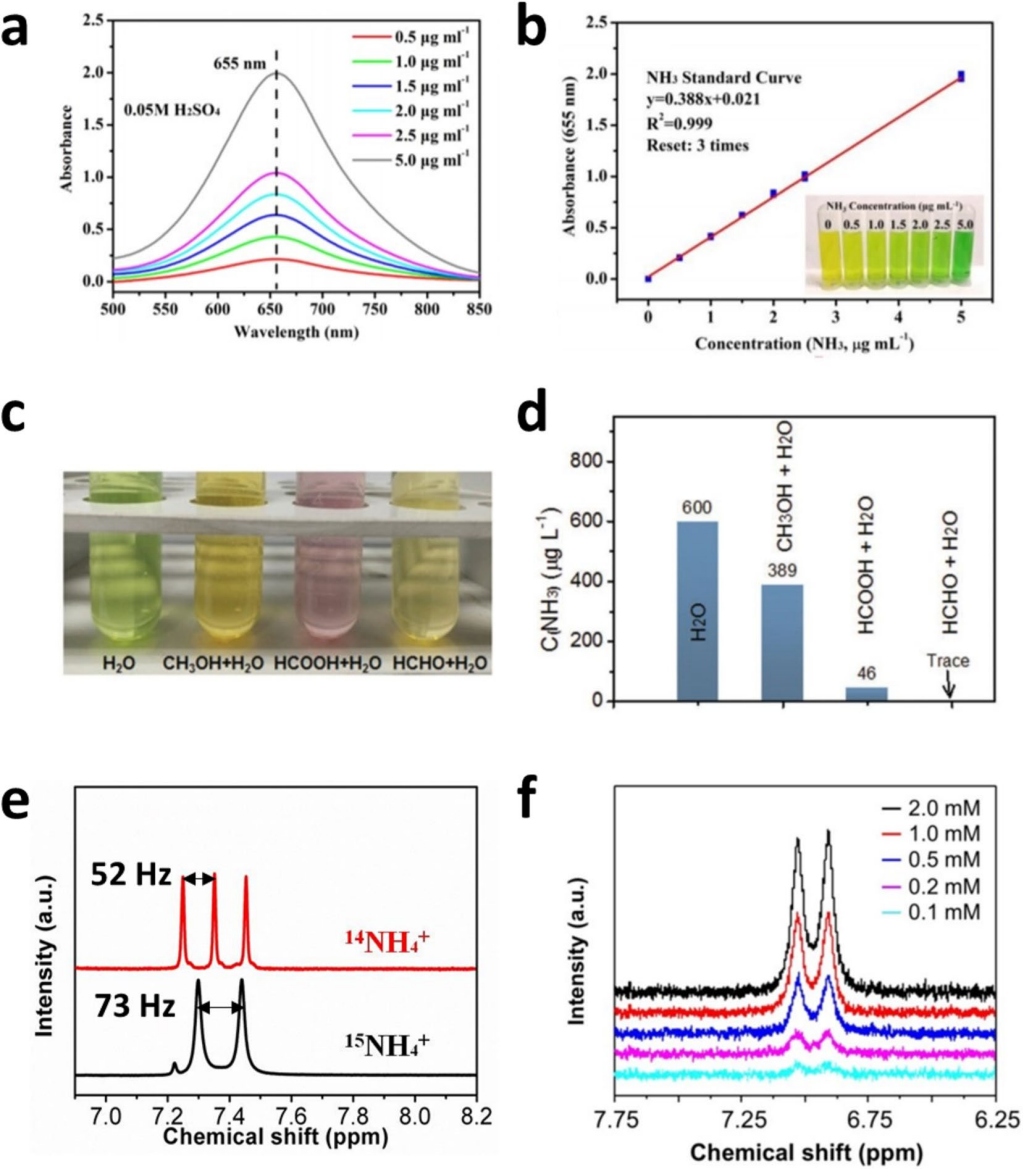
**a** Spectrum and **b** standard curve of UV–Vis spectrophotometer with standard solutions from 0.5 to 5.0 µg·mL^-1^ in 0.05 M H_2_SO_4_. Reproduced with permission [25]. Copyright 2019, PNAS. **c**, **d** Interference impact on measurements of produced NH_3_ in solution with CH_3_OH, HCOOH and HCHO. Reproduced with permission [26]. Copyright 2019, Wiley–VCH. **e** ^1^H-NMR spectrums of ^14^NH_4_^+^(red) and ^15^NH_4_^+^(black), respectively. Reproduced with permission [27]. Copyright 2021, The Royal Society of Chemistry. **f** The peaks for ^14^NH_4_^+^ corresponding to its concentration. Reproduced with permission [8]. Copyright 2020, American Chemical Society

The simplest photocatalytic NH_3_ production set-up is a particulate PC system where photocatalysts are dispersed in a medium, typically pure water, with N_2_ bubbling under simulated solar light illumination, as shown in Fig. 1b. NH_3_ and O_2_ are generated on a single photocatalyst. The produced NH_3_ is technically evaluated by the spectrophotometric method via the indophenol blue method or quantified using proton nuclear magnetic resonance spectroscopy (^1^H-NMR) with isotope labeled nitrogen ^15^ N as shown in Fig. 2.

The indophenol blue method is based on the principle of the Berthelot reaction, where typically, salicylic acid, citrate, hypochlorite, and ferricyanide are used in an alkaline solution. In the presence of NH_4_^+^, the color of the yellowish indicating solution, which contains the reagents, turns to blue, corresponding to 655 nm. As shown in Fig. 2a and b, thus, the concentration of NH_3_ can be quantified by measuring the absorbance change at 655 nm using a UV–Vis spectrophotometer [28]. However, to quantify the concentration of NH_3_ generated by photocatalytic N_2_ reduction, multiple possible contaminants that interfere with the evaluation should be fully controlled [28]. For example, methanol, a widely used as a hole scavenger, and its derivatives cause incorrect color changes and significantly decrease the accuracy of NH_3_ quantification as shown in Fig. 2c and d. Another critical point for NH_3_, which is photocatalytically produced, quantification is to eliminate the parts from NH_3_ contaminants, unintentionally present introduced through catalyst, components in set-up [26] or even the air. In fact, NH_3_ present in human breath, sanitizers, and so can rapidly accumulate in water [29].

The most rigorous procedure to quantify photocatalytically generated NH_3_ from N_2_ reduction is to use isotope labeled ^15^N_2_ gas. The photocatalytic reactions are conducted in a simple particulate system with ^15^N_2_ bubbling. Since the splitting of ^1^H resonance in ^15^NH_4_^+^ and ^14^NH_4_^+^ resulting from the difference in the scalar interaction between N (^15^ N or ^14^ N) and H is differentiated, the ^15^NH_4_^+^ produced, which is dissolved in an aqueous reacting solution, can be quantified by ^1^H-NMR [30]. For instance, the ^1^H resonance in ^15^NH_4_^+^ can be split into two symmetric signals with a spacing of 73 Hz, whereas the resonance in ^14^NH_4_^+^ can be split into three symmetric signals with a spacing of 52 Hz as shown in Fig. 2e. Thus, false positive evaluations for the produced NH_3_ can be prevented effectively.

## Strategies to develop photocatalysts

**Fig. 3:**
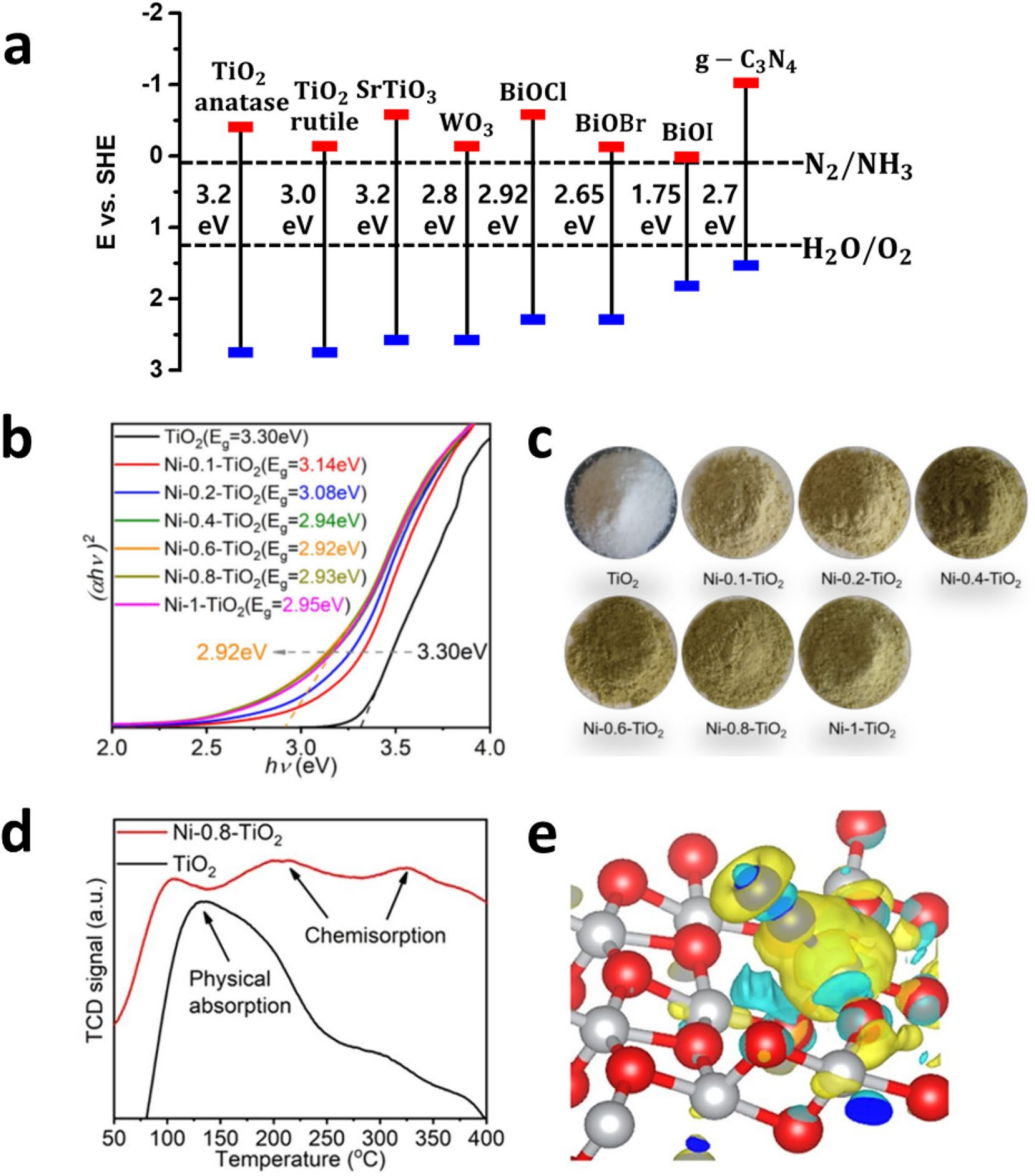
**a** The band alignments of widely used photocatalysts. **b** The Tauc plot (*ahv*)^2^ versus (*hv*) for the caluate the optical band gap energy E_g_. **c** The color of Ni-x-TiO_2_ gradually deepens with the increase of the amount of Ni^2+^. **d** The N_2_-TCD spectrums of Ni-0.8-TiO_2_(red) and TiO_2_(black), respectively. **e** Bader charge density difference of Ni doped TiO_2﻿_; Ti(silver), Ni(blue), and O(red). Reproduced with permission [45]. Copyright 2020, Americal Chemical Society

This section is divided into mainly two parts: (1) semiconductors comprising photocatalysts, which have been widely applied as a light absorber in Fig. 3a, and (2) strategies to modify the catalysts, which have been effectively used to further increase the photocatalytic NH_3_ production.

### Semiconductors

**Titanium(Ti)-based oxides** Titanium dioxide, TiO_2_, possesses several advantages that make it attractive for use in photocatalytic NH_3_ production. TiO_2_ has a bandgap of ~ 3.2 eV, which absorbs UV light, and a CBM and VBM that offer sufficient overpotential for the photoexcited electrons to reduce N_2_ to NH_3_ and holes to oxidize water to O_2_, respectively. In addition, the inexpensive, environmentally benign, and stable nature of TiO_2_ is an additional advantage. Historically, the primary limitation of using TiO_2_ has been rapid photoexcited electron–hole recombination, which is similar to many other oxide-based photocatalysts. Strontium titanate, SrTiO_3_ [31], is another candidate showing two major benefits over TiO_2_ for use in the NH_3_ production. The CBM of SrTiO_3_ is slightly more negative than that of TiO_2_, thereby providing a stronger driving force to reduce N_2_ using the photoexcited electrons. In addition, intrinsic charge mobility in SrTiO_3_ is higher than that in TiO_2_, thereby suppressing photoexcited charge recombination [32]. However, despite this thermodynamic advantage, the photocatalytic NH_3_ production using TiO_2_ or SrTiO_3_ is limited because of ineffective light absorption resulting from their wide bandgaps and inactive redox reactions due to the limited active sites on their surface. Recently, many attempts have been made to address these limitations, which include doping, coupling with electrocatalysts (cocatalysts), surface defect engineering, and so on [33–35].

**Bismuth oxyhalide (BiOX)** BiOX (X = Cl, Br, and I) has a layered structure where bismuth and oxygen layers (BiOx) are connected with halide (X) layers alternatingly via Van der Waals forces [36]. Since the VBM is determined by O 2p and X $\updelta$ p ($\updelta$ = 3, 4, and 5, corresponding to X = Cl, Br, and I, respectively) and the CBM is mainly evaluated by Bi 6p, their band alignments and bandgap are varied depending on the element comprising the semiconductors [37]. For example, BiOCl, BiOBr, and BiOI have bandgap energies of 2.92, 2.65, and 1.75 eV, respectively [38], and are thermodynamically suitable for NH_3_ production, they enable the production of NH_3_ and O_2_ by the overall photocatalytic N_2_ reduction. However, despite these advantages, BiOX semiconductors have severe drawbacks when using them as photocatalysts, mainly due to the insufficient photogenerated charge separation in the bulk and surface. Recently, their fundamental limitations have been investigated, and strategies to improve their photocatalytic performances have been explored.

**Graphitic carbon nitride (g-C**_**3**_**N**_**4**_**)** g-C_3_N_4_ is a polymeric semiconductor with a bandgap energy of 2.7 eV and appropriate positions for the CBM and VBM for the NH_3_ and O_2_ production, respectively [39]. However, since rapid photoexcited carrier recombination in the bulk results in limited charge separation in the redox reactions and ineffective N_2_ adsorption on the surface, as an initial step for N_2_ reduction, multiple advancements to develop g-C_3_N_4_ have been suggested [40].

### Strategies to enhance photocatalytic NH_3_ production

As described in the introduction section, photocatalysts comprising semiconductors can produce NH_3_ using N_2_ and water under illumination. However, the typical NH_3_ production rate and efficiency of intrinsic semiconductors using solar energy have been far lower than those expected from their bandgap energies. The major limiting factors include (1) limited light absorption because of the intrinsically large bandgaps, (2) significantly fast photoexcited electron–hole pairs recombination, and (3) slow redox reactions including N_2_ reduction and water oxidation. Recently, many strategies have been made to address one or more of these challenges, which include engineering defects by extrinsic doping or vacancy introducing, composites forming using multi-light absorbers, and coupling with electrocatalysts. This section will give an overview of each of these attempts and discuss how these strategies affected the photocatalytic NH_3_ production.

#### Defect engineering

Defect engineering has been widely suggested to control bulk and surface properties of catalysts, for example, by heteroatoms doping, vacancies formation, and so on. This defect engineering enhances photocatalytic NH_3_ production by changing the light absorption properties, improving charge separation efficiencies, or facilitating surface redox reactions, and it generally influences the multi-mechanistic steps of light harvesting simultaneously [41].

**Heteroatom doping** Heteroatom doping involves intentional introduction of impurity atoms into the lattices of materials to change their optical and electrical properties [42–44]. Depending on the species and degree (concentration) of dopants, changes, in the bandgap energy, band alignment, charge separation efficiency, and N_2_ adsorption ability are expected. However, since dopants can provide electron–hole recombination sites or prohibit charge transports, identifying types and concentrations of dopants appropriate for the photocatalytic process is important.

Zaicheng Sun and coworkers prepared a nickel-doped TiO_2_ (Ni-x-TiO_2_, where x = concentration of the Ni precursor controlled in the preparation step) photocatalyst using the sol–gel method [45] show Their optimized sample (Ni-0.8-TiO_2_) exhibited an NH_3_ production rate of 46.80 µmol·g^−1^·h^−1^ under simulated solar light irradiation, which is seven times higher than the rate using pure reference TiO_2_. The Ni-0.8-TiO_2_ sample extends the light absorption range to the visible light region with a bandgap energy of 2.92 eV, whereas pure TiO_2_ absorbs only UV light with a bandgap energy of 3.2 eV as shown in Fig. 3b. As Ni atoms having 2 + valence that replace the Ti site having 4 + valence, oxygen vacancies (Ov) are created naturally for charge neutrality within the host semiconductors, and they vary the band position of the CBM and VBM in TiO_2_, which results in a decrease in the bandgap energy. Besides changes in the optical properties, N_2_ adsorption, the initiation step for NH_3_ production from N_2_, is enhanced by Ni doping on TiO_2_, as investigated using a N_2_ temperature programmed desorption spectroscopy (N_2_-TPD) and a computational analysis.

Tierui Zhang and coworkers prepared a copper-doped TiO_2_ nanosheet (x%-TiO_2_, where x = molar ration of Cu/Ti controlled in the preparation step) photocatalyst using a hydrothermal method [46]. The optimized sample, 6%-TiO_2_, exhibited an NH_3_ production rate of 78.9 µmol·g^−1^·h^−1^, whereas pristine TiO_2_ nanosheets exhibited a rate of 0.34 µmol·g^−1^·h^−1^. As Cu heteroatoms replaced the sites of Ti in the TiO_2_ nanosheets, small amounts of Ti^3+^ valence states in Ti^4+^-dominant TiO_2_ and Ov sites are created, as observed using X-ray photoelectron spectroscopy and X-ray absorption fine structure spectroscopy. Furthermore, owing to the difference in size between the Cu^2+^ dopants and Ti^4+^ hosts, Cu-doped TiO_2_ has a compressive strain, which is evaluated by DFT calculations, and it causes changes in the distribution in the electron densities around O and Ti in the materials. The combination of non-stoichiometry and lattice strain in Cu-doped TiO_2_ narrows its bandgap energy, corresponding to the extended light absorption range of 600–800 nm, and electrons are accumulated around the sites of O atoms, thereby affecting the changes in the N_2_ adsorption affinity.

Jing Zhang and coworkers prepared a Fe-doped SrTiO_3_ (Fe_x_Sr_1-x_TiO_3_, where x = the stoichiometric ratio of Fe precursor and Sr precursor controlled in preparation step) photocatalyst using the hydrothermal method followed by calcination [47]. The Fe_0.1_Sr_0.9_TiO_3_ sample exhibited the best NH_3_ production rate of 30.1 μmol·g^−1^·h^−1^, which is 3.2 times higher than that of pristine SrTiO_3_. By replacement of the Fe^3+^ ion, which has a smaller size, on the Sr^2+^ site in SrTiO_3_, the size of particles is reduced along with the corresponding increase in the surface area. Furthermore, the Fe_0.1_Sr_0.9_TiO_3_ photocatalyst exhibits significantly enhanced N_2_ chemisorption and activation ability, which Fe dopants are the major contributor to.

**Vacancies** Vacancy, one of defect formation strategies, leads to changes in the band structure and chemical adsorption nature on the surface [48]. This section gives an overview of intentionally induced anion vacancies in semiconductor photocatalysts and discusses how they affected the photocatalytic NH_3_ production.

**Fig. 4:**
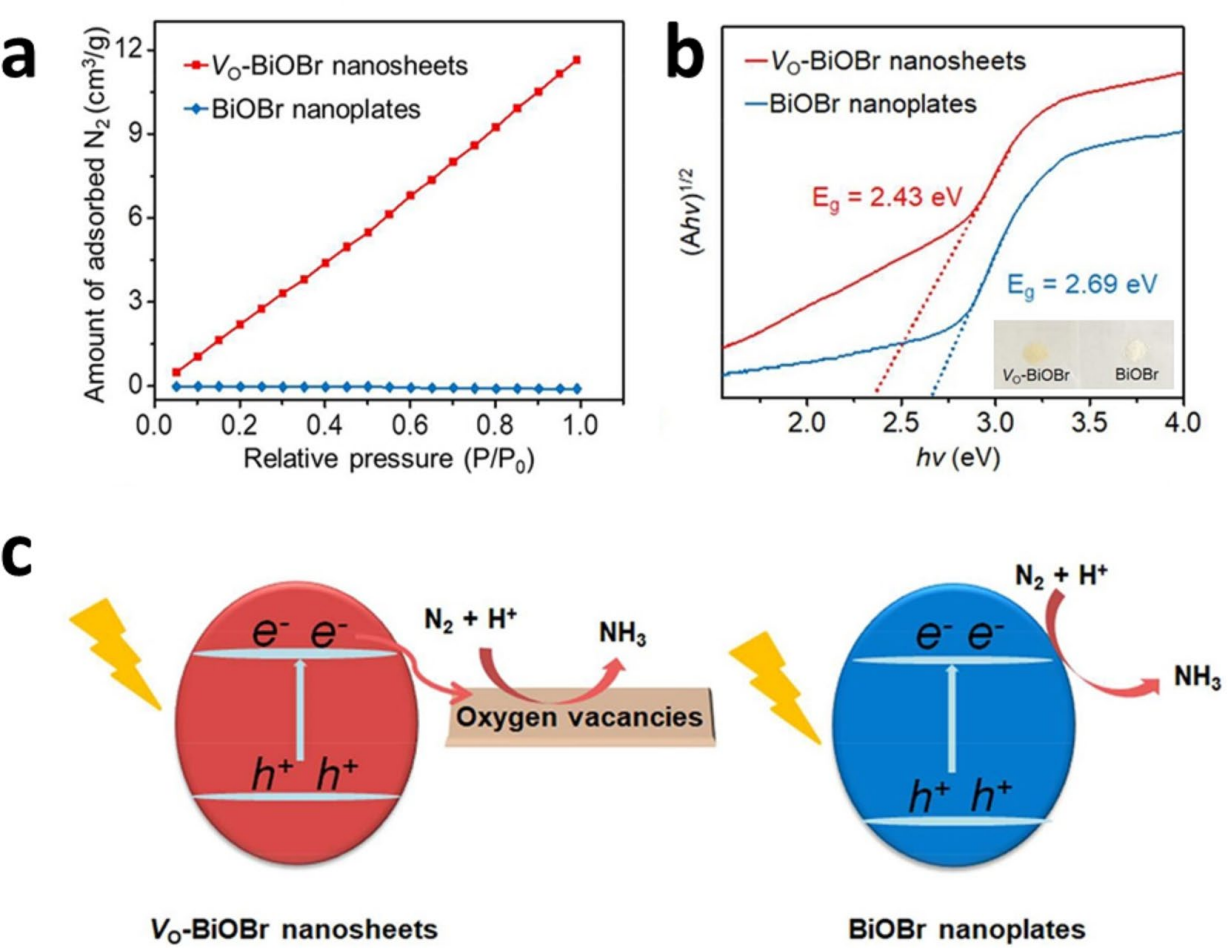
**a** The N_2_ adsorption isotherms, **b** The Tauc plot (*ahv*)^2^ versus (*hv*) and **c** The schematic illustration of pohtocatalytic NH_3_ synthesis process on Vo-BiOBr nanosheets and BiOBr nanoplates, respectively. Reproduced with permission [49]. Copyright 2018, American Chemical Society

Zhong Jin and coworkers compared the NH_3_ production activities using BiOBr semiconductors in the presence (Vo–BiOBr) or absence (BiOBr) of oxygen vacancies [49]. The Vo-BiOBr samples were prepared using the hydrothermal method with the addition of polyvinylpyrrolidone surfactants leading to in-situ generation of abundant vacancies, whereas pristine BiOBrs were synthesized without adding the surfactants. The Vo-BiOBr photocatalysts produced NH_3_ at a rate of 54.70 μmol·g^−1^·h^−1^ under UV–Vis irradiation, which is 10 times higher than the production rate using BiOBr. The major contribution of this significant improvement is enhanced N_2_ adsorption that initiates the intermediate formation for NH_3_ production. For example, the amount of adsorbed N_2_, estimated based on N_2_ adsorption isotherms, on Vo-BiOBr is considerably higher than that using BiOBr as shown in Fig. 4a. Furthermore, by introduction of the oxygen vacancies, the bandgap energy is reduced with the shifts in the CBM and VBM positions, which leads to increased light absorption as shown in Fig. 4b.

Chuanyi Wang and coworkers prepared metal-free g-C_3_N_4_ semiconductors by thermal decomposition of melamine and induced nitrogen vacancies (V-g-C_3_N_4_) within the semiconductors by additional calcination under a N_2_ atmosphere [50]. The V-g-C_3_N_4_ samples produced NH_3_ at a rate of 1240 μmol·g^−1^ h^−1^ using pure N_2_ and water under visible light irradiation, whereas pristine g-C_3_N_4_ produced negligible amounts of NH_3_. Further, morphologies and bandgap energies were not significantly varied depending on the intentional creation of N vacancies; however, two factors changed considerably. Due to the presence of N vacancies on the semiconductor, the signal collected using photoluminescence spectra decreased, indicating the reduction in the carrier recombination and enhancement of their separation. Furthermore, the N vacancies enable selective adsorption and activate N_2_, and it was experimentally confirmed that negligible amount of NH_3_ produced on g-C_3_N_4_ selectively blocked the vacancies using Pd particles.

#### Coupling with multi-light absorbers

Coupling with multi-light absorbers to form heterojunctions has been widely investigated to improve light absorption capacity under single illumination and separation of photoexcited electron–hole carriers through their designed band alignments. This strategy for catalyst design is effective in improving the photocatalytic NH_3_ production.

**Fig. 5:**
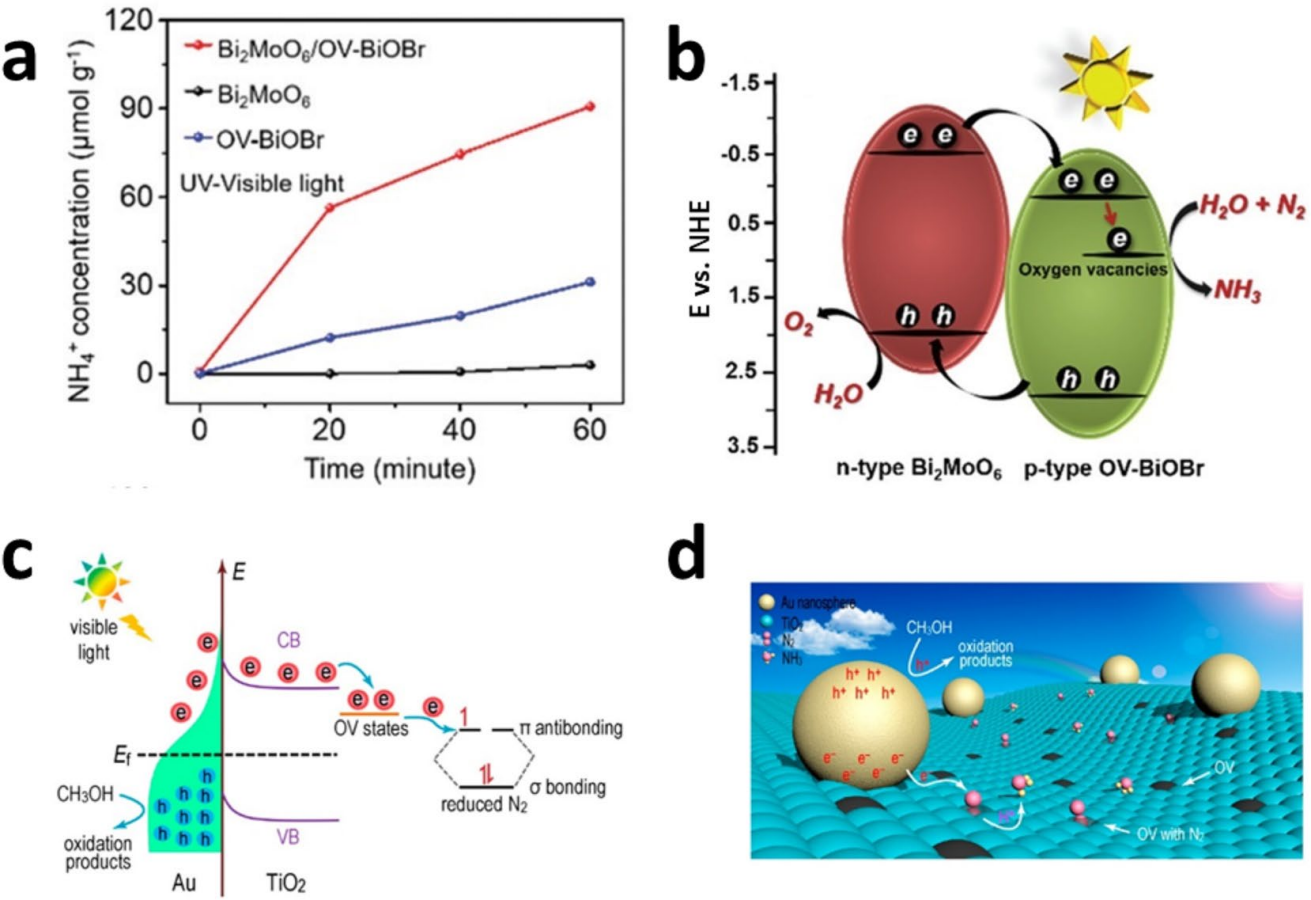
**a** NH_4_^+^ concentrations of Bi_2_MoO_6_/Ov-BiOBr heterojunctions, Bi_2_MoO_6_ and BiOBr alone, respectively. **b** The scheme of proposed Bi_2_MoO_6_/BiOBr heterojunctions. Reproduced with permission [51]. Copyright 2019, The Royal Society of Chemistry. **c** The schematic reaction mechanism and **d** the artistic illustration of Au/TiO_2_-Ov photocatalyst. Reproduced with permission [52]. Copyright 2018, American Chemical Society

Zhong Jin and coworkers prepared Bi_2_MoO_6_/Ov-BiOBr composites, where Ov denotes the oxygen vacancies using two-step methods, and synthesized Bi_2_MoO_6_ by solution-phase reflux process followed by the hydrothermal process to couple Ov-BiOBr on as-prepared Bi_2_MoO_6_ [51]. Despite minimal difference in light absorption before and after coupling, the Bi_2_MoO_6_/Ov-BiOBr composites showed a significantly enhanced NH_3_ production rate (90.7 μmol·g^−1^ h^−1^), which is almost 30 and 3 times higher than the rates evaluated using Bi_2_MoO_6_ and Ov-BiOBr, respectively as shown in Fig. 5a. The major role of coupling is the formation of cascaded band alignments at the interface between two semiconductors. The photoexcited holes are transported and oxidize the electron donor on Bi_2_MoO_6_ where the VBM is located relatively higher, whereas the photoexcited electrons are transported and reduce N_2_ on Ov-BiOBr where the CBM located relatively lower. Furthermore, oxygen vacancies on BiOBr show synergistic effects on the NH_3_ production by providing N_2_ adsorption and activation sites as shown in Fig. 5b.

Jimmy Yu and coworkers prepared Ov-TiO_2_ using the hydrothermal method followed by loading Au nanoparticles. The Au nanoparticles generate hot electrons under visible light illumination [51], which is known as plasmonic phenomena. The hot electrons are injected into the CBM of TiO_2_ and then trapped in vacant sites where N_2_ is reduced into NH_3_. The generated holes remain on Au and oxidize the electron donors, methanol, in solution as shown in Fig. 5c and d. The optimized Au/TiO_2_-Ov samples produced NH_3_ with a rate of 78.6 μmol·g^−1^·h^−1^ under visible light irradiation, which is at least 98 and 35 times higher those using Au/TiO_2_ and TiO_2_-Ov photocatalysts, respectively.

#### Surface modification and reaction engineering

As discussed above, photocatalytic activities have been developed significantly by modifying photocatalysts, for example, defects engineering and composite formation. However, although photogenerated charge generation and separation are advanced by multiple possible strategies, if the surfaces of the semiconductors exhibit slow charge injection and are poorly catalytic for N_2_ reduction, the NH_3_ production rate may not be enhanced directly. The surface modification on the semiconductors using a robust electrocatalyst (cocatalyst) for N_2_ to NH_3_ conversion is the simplest way to improve the limiting factor at the interface between the semiconductors and liquid where reactants are present. Therefore, the modification of the semiconductor surface with various NH_3_ production catalysts, such as Ru, Cu, and Au, has been investigated [20, 34, 53].

In contrast, since the rates of reduction and oxidation reactions always correspond to one another, the sluggish photo-oxidation using holes may become a limiting factor for the photocatalytic NH_3_ production. To overcome the possible limitation, it has been widely suggested to load appropriate electrocatalysts (cocatalysts) for water oxidation on the semiconductor or provide a more facile hole acceptor (electron donors) than water, such as methanol or ethanol (Table 1).

**Table 1:** The summary of photocatalytic nitrogen reduction performances and its operating conditions: photocatalysts

Photocatalysts	Light source	Hole scavenger	Ammonia production rate	Ammonia detection methods	Refs
PCN/rGO(0.2)	250 W Xe lamp 400 nm < λ < 800 nm	1 mM EDTA-2Na	544.6 µmol·L^-1^·g^−1^·h^−1^	Nessler’s reagent^b^	[14]
Ru/TiO_2_	300 W Xe lamp	20% C_2_H_5_OH	3.31 µmol·g^−1^·h^−1^	Indophenol blue	[20]
Cu/g-C_3_N_4_	300 W Xe lamp 400 nm < λ < 800 nm	20% C_2_H_5_OH	186 µmol·g^−1^·h^−1^	Nessler’s reagent	[34]
Ni-0.8-TiO_2_	300 W Xe lamp	None	46.80 µmol·g^−1^·h^−1^	Nessler’s reagent	[45]
6%-TiO_2_	300 W Xe lamp	None	78.9 µmol·g^−1^·h^−1^	Nessler’s reagent	[46]
Fe_0.1_Sr_0.9_TiO_3_	300 W Xe lamp	None	30.1 µmol·g^−1^·h^−1^	Nessler’s reagent	[47]
Vo-BiOBr nanosheets	300 W Xe lamp	None	54.70 µmol·g^−1^·h^−1^	Nessler's reagent	[49]
V-g-C_3_N_4_^a^	300 W Xe lamp 420 nm > λ	20% CH_3_OH	1240 µmol·g^−1^·h^−1^	Nessler’s reagent	[50]
Bi_2_MoO_6_/Ov-BiOBr	300 W Xe lamp	None	90.7 µmol·g^−1^·h^−1^	Nessler’s reagent	[51]
Au/TiO_2_-Ov	300 W Xe lamp 420 nm > λ	10% CH_3_OH	78.6 µmol·g^−1^·h^−1^	Indophenol blue	[52]
Au/(BiO)_2_CO_3_	300 W Xe lamp	None	38.2 µmol·g^−1^·h^−1^	Indophenol blue	[53]
BiOCl	500 W Xe lamp	25% CH_3_OH	92.4 µmol·g^−1^·h^−1^	Nessler's reagent	[54]
Bi_5_O_7_I	300 W Xe lamp	20% CH_3_OH	111.5 µmol·L^−1^·h^−1^	Nessler’s reagent	[55]
Bi_5_O_7_Br	300 W Xe lamp 400 nm > λ	None	1380 µmol·g^−1^·h^−1^	Nessler's reagent	[56]
20Fe-2Al@3DGraphene	500 W Hg lamp	None	430 µmol·g^−1^·h^−1^	Indophenol blue	[57]
Ultrathin MoS_2_	500 W Xe lamp 420 nm > λ	None	325 µmol·g^−1^·h^−1^	Isotopic labeling	[58]
CuCr-NSs	300 W Xe lamp 400 nm > λ	None	184.8 µmol·L^−1^·h^−1^	Nessler’s reagent	[59]
Mo-doped W_18_O_49_	300 W Xe lamp	1 mM Na_2_SO_3_	195.5 µmol·g^−1^·h^−1^	Nessler’s reagent, Ion chromatography	[60]
BiO quantum dots	500 W Xe lamp	None	1226 µmol·g^−1^·h^−1^	Indophenol blue	[61]
C-WO_3_·H_2_O	500 W Xe lamp	None	205 µmol·g^−1^·h^−1^	Nessler's reagent	[62]
Fe-doped g-C_3_N_4_	250 W Na lamp 400 nm < λ < 800 nm	0.1% C_2_H_5_OH	317.1 µmol·L^−1^·g^-1^·h^−1^	Nessler’s reagent	[63]
5% Ru@n-GaN NWs	300 W Xe lamp	None	120 µmol·g^−1^·h^−1^	Indophenol blue	[64]
Ga_2_O_3_-DBD/g-C_3_N_4_	500 W Xe lamp	0.04 mM CH_3_OH	112.5 µmol·L^−1^·h^−1^	Nessler’s reagent	[65]
TiO_2_@C/g-C_3_N_4_	300 W Xe lamp 420 nm > λ	20% CH_3_OH	250.6 µmol·g^−1^·h^−1^	Nessler’s reagent	[66]
g-C_3_N_4_/MgAlFeO	250 W Na lamp 400 nm < λ < 800 nm	0.1% C_2_H_5_OH	440.4 µmol·L^-1^·g^−1^·h^−1^	Nessler's reagent	[67]
TiO_2_/Au/a-TiO_2_	300 W Xe lamp	None	0.0134 µmol·cm^−2^·h^−1^	Indophenol blue	[68]
Fe_2_O_3_	500 W Xe lamp	0.1% C_2_H_5_OH	1362.5 µmol·L^−1^·h^−1^	Nessler’s reagent	[69]
H-Bi_2_MoO_6_	300 W Xe lamp with a 420 nm cutoff filter	None	1300 µmol·g^−1^·h^−1^	Nessler’s reagent	[70]

^a^Where V denoted nitrogen vacancies

^b^Nessler’s reagent, K_2_HgI_4_, is one of photometric indicators for NH_3_ detection and quantification [71]

## Summary and outlook

In summary, we reviewed the mechanistic and experimental steps for photocatalytic NH_3_ production and examined various strategies to modify photocatalysts employed to enhance the production activities. This review clearly shows that although significant development of photocatalyts has been reported thus far, further advances for practical NH_3_ production are required. First, the efficiency for charge generation via solar light absorption should be enhanced. Considering that the majority of incident photons are in the range of visible light, development of absorbers, can harvest the incident solar light, is required by determining appropriate materials and engineering semiconductors.

The charge separation effect is also required for photocatalytic NH_3_ production through suppression of their recombination. For example, strategies for doping and composite formation will exhibit advances in the future and may increase the photon absorption efficiency simultaneously. In addition, morphology engineering [72–76] for photocatalysts will be promising and can be achieved by controlling the distances for carrier transportation and lifetime duration between the photo-induced generation and recombination. Furthermore, this may change facets and surface area, thereby providing active sites for the redox reactions using transported carriers. Moreover, if the facets are optimized with increasing surface area, morphology engineering can be significantly beneficial for NH_3_ production.

With improvements in photon absorption and charge separation, surface engineering will be able to provide active sites where N_2_ reduction using electrons and oxidation using holes is important. Thus, defect engineering, for example, formation of vacancies, cocatalyst coupling, and applying sacrificial reagents have been suggested. It is noteworthy that N_2_ diffusivity control close to photocatalysts make an important contribution to practical photocatalytic NH_3_ production. For instance, importance of three-phase interfaces, where a catalyst and two phases reactants such as water and active gas faced, has been demonstrated [77,78]. This indicates that even if highly robust catalysts are prepared, if the supply of N_2_ gas and water is insufficient at the region close to the catalyst, the NH_3_ production is limited. Thus, environmental engineering, for example, via hydrophobicity control on the catalysts, will be significantly effective. The mechanism, experimental methods, discussion, insights, and suggestions contained in this review provide a good fundamental and foundation for further improvement of photocatalytic NH_3_ production.

## Data Availability

The datasets used and analysed during the current study are available from the corresponding references listed.
